# IGFBP-rP1 suppresses epithelial–mesenchymal transition and metastasis in colorectal cancer

**DOI:** 10.1038/cddis.2015.59

**Published:** 2015-03-19

**Authors:** S Zhu, J Zhang, F Xu, E Xu, W Ruan, Y Ma, Q Huang, M Lai

**Affiliations:** 1 Department of Pathology, School of Medicine, Zhejiang University, Hangzhou, Zhejiang, China; 2 Key Laboratory of Disease Proteomics of Zhejiang Province, Hangzhou, Zhejiang, China

## Abstract

Epithelial–mesenchymal transition (EMT) was initially recognized during organogenesis and has recently been reported to be involved in promoting cancer invasion and metastasis. Cooperation of transforming growth factor-*β* (TGF-*β*) and other signaling pathways, such as Ras and Wnt, is essential to inducing EMT, but the molecular mechanisms remain to be fully determined. Here, we reported that insulin-like growth factor binding protein-related protein 1 (IGFBP-rP1), a potential tumor suppressor, controls EMT in colorectal cancer progression. We revealed the inhibitory role of IGFBP-rP1 through analyses of clinical colorectal cancer samples and various EMT and metastasis models *in vitro* and *in vivo*. Moreover, we demonstrated that IGFBP-rP1 suppresses EMT and tumor metastasis by repressing TGF-*β*-mediated EMT through the Smad signaling cascade. These data establish that IGFBP-rP1 functions as a suppressor of EMT and metastasis in colorectal cancer.

Colorectal cancer (CRC) is one of the most common human malignancies, accounting for nearly 1 in 10 cancer deaths.^1^ More than 90% of these deaths are attributed to local invasion and distant metastasis.^2^ Therefore, it is of great importance to understand the underlying biological processes. Compelling evidence suggests that epithelial–mesenchymal transition (EMT) is a major mechanism of cancer cell metastasis by endowing cells with an invasive phenotype.^3, 4^

EMT is a highly conserved cellular process that induces epithelial cells to dissociate from the primary tumor, invade surrounding tissues, and migrate to distant organs.^5, 6^ During EMT, cells lose their epithelial features of polarity and adhesion, gain mesenchymal properties, and exhibit increased motility.^7^ Biochemically, epithelial markers such as the adherens junction molecules E-cadherin and zonula occludens-1 (ZO-1) are repressed and mesenchymal markers such as vimentin and N-cadherin are upregulated, together with increased matrix metalloproteinase-9 secretion to degrade the extracellular matrix.^8, 9^ In addition, transcription factors such as members of the Snail (SNAIL and SLUG), ZEB (ZEB1 and SIP1), and bHLH (E47 and TWIST) families also contribute to the occurrence of EMT by binding to the E-boxes at the E-cadherin promoter to silence its expression.^10^

A variety of extracellular signals can trigger EMT, and transforming growth factor *β* (TGF-*β*) is considered to be a potent inducer of EMT in an autocrine or paracrine manner.^11^ It is known that TGF-*β* has dual roles during tumor progression. It inhibits tumor growth in the early phase, but paradoxically, in the late phase it acts as a tumor promoter by enhancing EMT and promoting tumor cell invasion in primary carcinomas.^12, 13^ TGF-*β* binds to its receptors and activates Smad2 and Smad3, then phosphorylated SMAD2/3 partner with Smad4 to translocate into the nucleus where SMAD complexes control target gene transcription, including EMT- and motility-related genes.^14^

Insulin-like growth factor-binding protein-related protein 1 (IGFBP-rP1, also known as IGFBP7) is a secreted factor belonging to the IGFBP family. Mounting evidence indicates that IGFBP-rP1 possesses tumor-suppressor activity in various tumor types.^15, 16, 17, 18, 19^ Indeed, our previous studies clearly revealed that IGFBP-rP1 inhibits cell proliferation, decreases soft agar colony formation, and induces apoptosis in CRC cell lines. Moreover, the abundance of IGFBP-rP1 is inversely correlated with a poor prognosis.^20^ In addition, when we restored the expression of IGFBP-rP1 with 5-aza-2′-deoxycytidine, the migration and invasion abilities of CRC cells decreased.^21^ In aggregate, although IGFBP-rP1 has been confirmed as a master regulator of cancer-cell fate, its functional role in EMT and metastasis remains largely unknown.

Here, we used patient samples and several CRC cell models to reveal a unique role for IGFBP-rP1 in inhibiting EMT and metastasis *via* a TGF-*β*/smad-dependent pathway.

## Results

### Negative correlation of IGFBP-rP1 detected by immunohistochemical staining with EMT features in clinical specimens

Considering the proposed correlation between IGFBP-rP1 and clinical outcome, we extended our analysis to the relationship between IGFBP-rP1 expression and clinicopathological parameters. The staining intensity of IGFBP-rP1 was inversely correlated with histological grade (*r*=−0.161, *P*=0.017), lymph-node metastasis (*r*=−0.174, *P*=0.011), and TNM stage (*r*=−0.149, *P*=0.028) (Table 1). Then, we investigated whether IGFBP-rP1 was also associated with EMT marker expression in our clinical cohort. Immunostaining of IGFBP-rP1 and the epithelial markers E-cadherin (*r*=0.24, *P*=0.014) and *β*-catenin (*r*=0.28, *P*=0.002) revealed a positive correlation in membrane expression. Conversely, we found a negative correlation between IGFBP-rP1 and fibronectin expression (*r*=−0.196, *P*=0.030) (Table 2). Overall, our clinical results imply that IGFBP-rP1, as a suppressor of CRC progression, may function to oppose the EMT program.

### Overexpression of IGFBP-rP1 hinders EMT and cell migration in CRC cells

**Figure 1:**
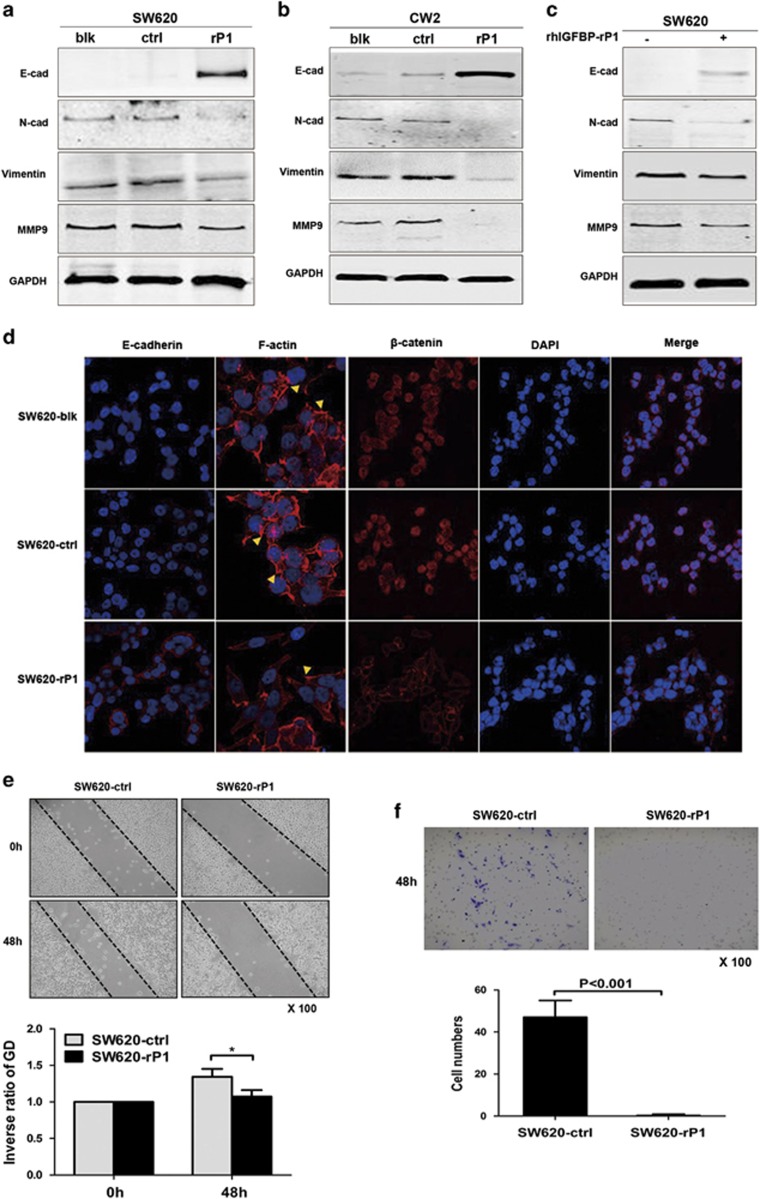
IGFBP-rP1 overexpression blocks EMT in CRC. (**a** and **b**) Well-established EMT markers were analyzed by western blot after IGFBP-rP1 transfection in SW620 and CW2 cells. E-cadherin expression was markedly induced and the mesenchymal markers N-cadherin, vimentin, and MMP9 were decreased in IGFBP-rP1-transfected cells (-rP1) compared with control vector-transfected (-ctrl) and parental cells (-blk). Equal loading was confirmed by GAPDH. (**c**) SW620 cells were treated with rhIGFBP-rP1 protein (4 μg/ml) for 48 h and the expression of EMT markers was detected by western blot. (**d**) Immunofluorescence images of SW620 cells stained for E-cadherin, *β*-catenin, and F-actin. The images were taken at × 630 (for E-cadherin and *β*-catenin ) and × 1000 (for F-actin). Arrow: Lamellipodia and microspike formation. DAPI and *β*-catenin staining was showed seperately and then the merged images were showed. (**e** and **f**) Wound-healing and transwell motility assays for SW620-ctrl and SW620-rP1 cells. The motility was drastically decreased in IGFBP-rP1 transfected SW620 cells ( × 100). Cell motility detemined by wound-healing assay was quantified as an inverse ratio of gap distance (GD) at 48 h relative to that at 0 h. *P< 0.05. The data were expressed as mean+S.D. of three independent experiments

To directly investigate the potential role of IGFBP-rP1, we stably overexpressed it in CRC cell lines and evaluated its effect on EMT. We found that IGFBP-rP1 overexpression increased the expression levels of the epithelial cell marker E-cadherin and blocked that of the mesenchymal markers N-cadherin, vimentin, and MMP9 in SW620 and CW2 cells (Figures 1a and b). As IGFBP-rP1 is a secreted protein, we sought to determine whether exogenous IGFBP-rP1 similarly changed EMT marker protein levels. In agreement with the changes in IGFBP-rP1-overexpressing cells, addition of recombinant human IGFBP-rP1 protein (rhIGFBP-rP1) resulted in high E-cadherin and low mesenchymal markers levels in SW620 cells (Figure 1c). Meanwhile, upregulated E-cadherin accumulated in the cell-to-cell junctions, and nuclear *β*-catenin re-localized to the membrane and cytoplasm of SW620 cells, as expected (Figure 1d). Furthermore, SW620-IGFBP-rP1 cells exhibited organized F-actin fibers as well as reduced stress fibers and lamellipodia at the periphery compared with the control cells and the parental cells (Figure 1d). Functionally, overexpression of IGFBP-rP1 decreased the motility ability (Figures 1e and f) of SW620 cells. Collectively, these data indicate that IGFBP-rP1 is a negative regulation of EMT in CRC.

### Knockdown of IGFBP-rP1 induces EMT and promotes invasion in CRC cells

**Figure 2:**
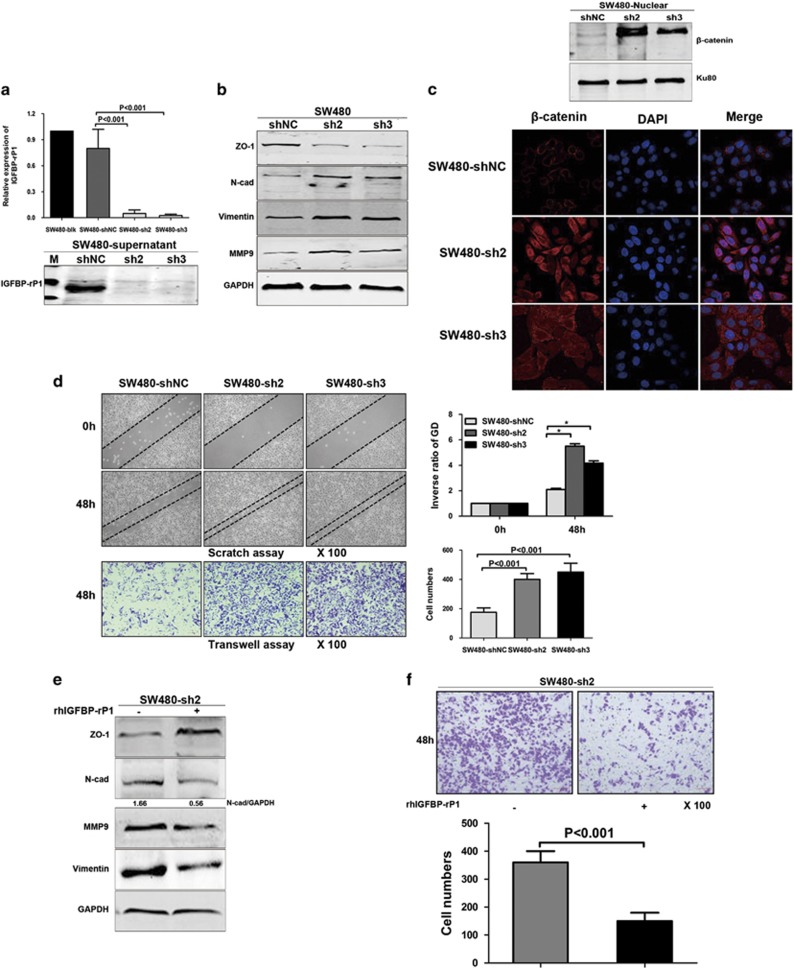
IGFBP-rP1 knockdown promotes EMT in SW480 cells. (**a**) The effectiveness of shRNA interference was confirmed by real-time RT-PCR and western blot analyses. The data were expressed as mean+S.D. of three independent experiments. (**b**) The expression of EMT markers was detected by western blot in scrambled control cells (shNC) and two stable IGFBP-rP1 knockdown cell clones (sh2 and sh3). (**c**) Nuclear expression of *β*-catenin detected by western blot in two SW480/shIGFBP-rP1 cell lines. Relocalization of *β*-catenin from adherens junctions of the membrane to cytoplasm and nucleus detected by immunofluorescence. (**d**) Wound-healing and transwell motility assays for SW480 cells expressing shRNA directed against IGFBP-rP1 or scrambled control shRNA ( × 100). *P<0.05. The data were expressed as mean+S.D. of three independent experiments. (**e** and **f**) The addition of rhIGFBP-rP1 protein to stable IGFBP-rP1-knockdown cells. SW480-sh2 cells were treated with rhIGFBP-rP1 protein (1 μg/ml) for 48 h and the expression of EMT markers was detected by western blot. Transwell motility assay was performed in SW480-sh2 cells treated with rhIGFBP-rP1 ( × 100) and value was shown as mean+S.D. of three independent experiments

We next determined whether knockdown of IGFBP-rP1 reversed its inhibitory role in EMT. We used short-hairpin RNA (shRNA) to stably knock down IGFBP-rP1 in SW480 cells, which have detectable endogenous IGFBP-rP1 expression (Figure 2a). To rule out clone-to-clone variations, two clones named SW480-sh2 and SW480-sh3 were selected. The knockdown efficiency of IGFBP-rP1 was further confirmed at the mRNA and protein levels (Figure 2a). IGFBP-rP1 downregulation induced EMT, leading to augmented N-cadherin, vimentin, and MMP9 protein levels as well as a significant decrease in ZO-1 expression (Figure 2b). Nuclear expression of *β*-catenin detected by western blot exhibited increased nuclear *β*-catenin levels in two SW480/shIGFBP-rP1 cell lines. Similarly, immunofluorescence showed that knockdown of IGFBP-rP1 results in relocalization of *β*-catenin from adherens junctions of the membrane to cytoplasm and nucleus (Figure 2c). Functionally, knockdown of IGFBP-rP1 dramatically increased the migration ability of SW480 cells (Figure 2d). In a complementary experiment, we determined whether the re-expression of IGFBP-rP1 rescued the EMT process induced by silencing it, and found that addition of rhIGFBP-rP1 protein restored ZO-1 expression as well as rescued the increased Vimentin, N-cadherin, and MMP9 expression triggered by IGFBP-rP1 knockdown in SW480 cells (Figure 2e). Consistently, the elevated transwell migratory cells were also reduced due to the restoration of IGFBP-rP1 (Figure 2f). Taken together, the functional role of IGFBP-rP1 in inhibiting EMT in CRC is further strengthened.

### IGFBP-rP1 is involved in tumorigenicity of CRC cells in nude mice

**Figure 3:**
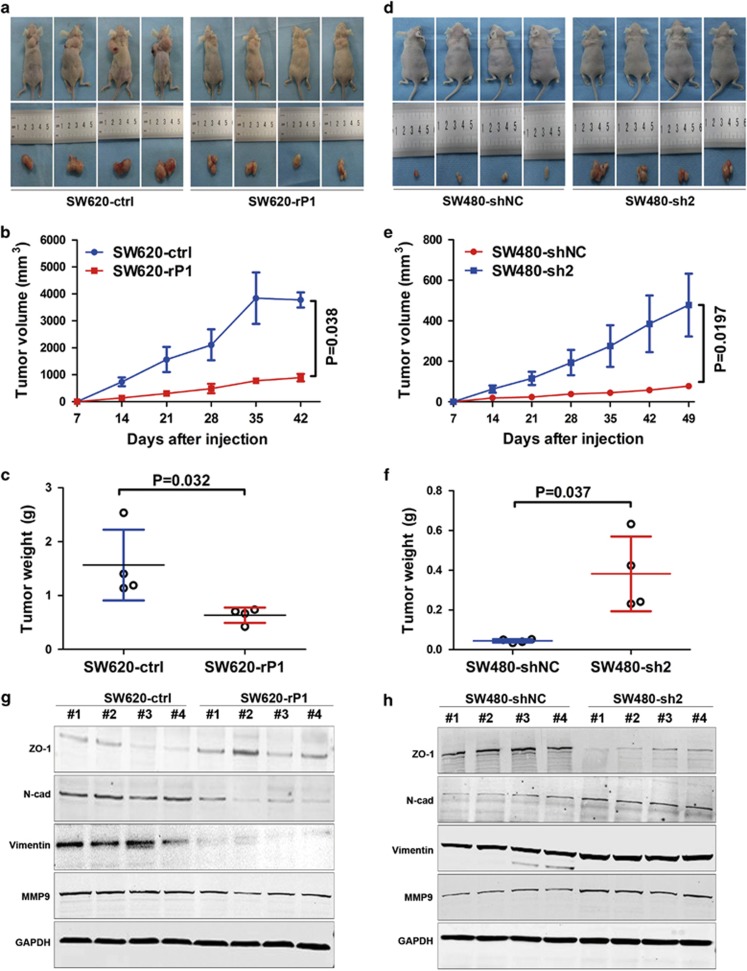
Tumorigenicity assay in nude mice. (**a**) SW620-ctrl and SW620-rP1 cells were injected subcutaneously into nude mice (*n*=4). Photographs of the tumors and mice were taken at the end of the study. (**b**) IGFBP-rP1 overexpression showed a lower growth rate (Mann–Whitney *U*-test). Points and bars represent the average±S.D. (**c**) Weights of xenograft tumors with and without IGFBP-rP1 overexpression. (**d**) Photographs of tumors and nude mice after SW480-sh2 and SW480-shNC cells were injected subcutaneously (*n*=4). (**e**) IGFBP-rP1 knockdown increased the tumor growth rate in nude mice (Mann–Whitney *U*-test). Points and bars represent the average±S.D. (**f**) The average tumor was heavier in the SW480-sh2 group than the SW480-shNC group. (**g** and **h**) EMT markers examined by western blot in harvested mouse tumor samples (GAPDH as a loading control)

To explore the biological role of IGFBP-rP1 in tumor formation *in vivo*, we inoculated CRC cell lines with and without IGFBP-rP1 expression as xenografts in nude mice. In accord with the finding that IGFBP-rP1 inhibited cell proliferation *in vitro*, the xenografted tumors in the IGFBP-rP1 overexpressing SW620 group were smaller, both in size (Figures 3a and b) and in weight (Figure 3c), than those formed by control cells. Conversely, the tumors formed by IGFBP-rP1 knockdown SW480 cells were larger (Figures 3d and e) and heavier (Figure 3f) than the control tumors. To characterize the EMT features of IGFBP-rP1-induced CRC tumors, we performed western blot analyses on all xenograft tumors. Consistent with the *in vivo* findings, we noted that IGFBP-rP1 overexpression increased the epithelial marker ZO-1 and reduced the mesenchymal markers N-cadherin, vimentin, and MMP9 (Figure 3g), whereas silencing of IGFBP-rP1 had the contrary effect (Figure 3h). These findings support an inhibitory role of IGFBP-rP1 in the EMT *in vivo*.

### IGFBP-rP1 inhibits colorectal cancer metastasis

**Figure 4:**
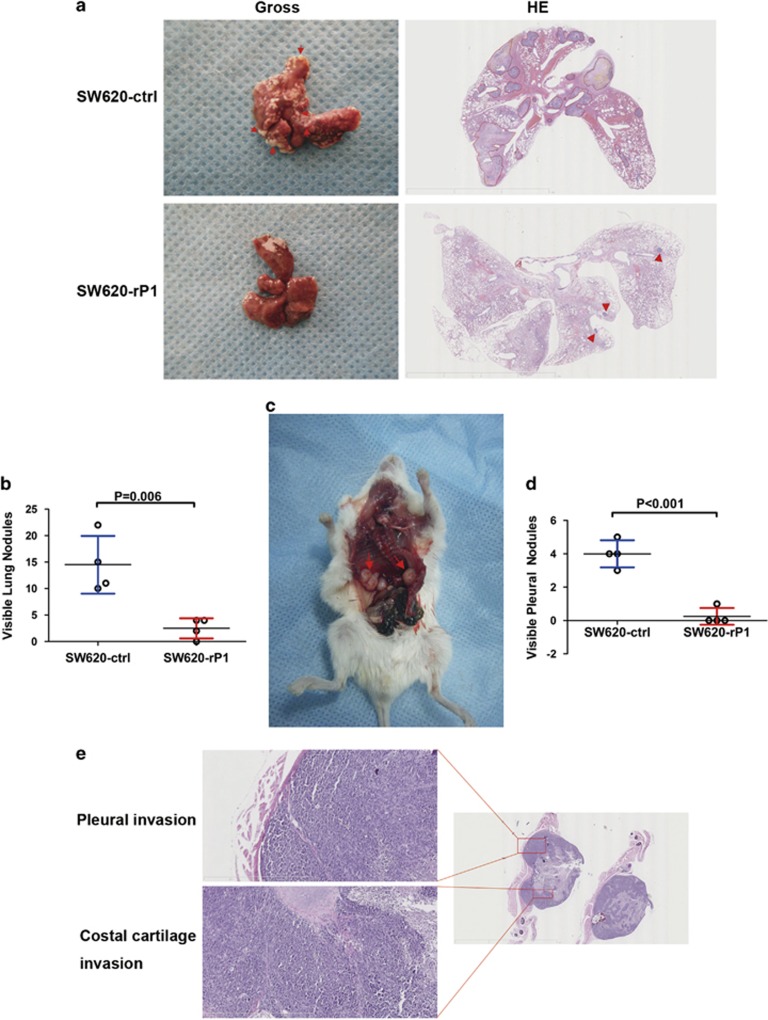
IGFBP-rP1 inhibited tumor metastasis in NOD/SCID mice. (**a**) Representative gross images and hematoxylin and eosin-stained lung sections from animals injected intravenously with control or IGFBP-rP1 overexpressing SW620 cells. Arrows, curve, and triangle: metastatic nodes in the lung. Scale bars, 9 mm (right top) and 8 mm (right bottom). (**b**) Box plot showing the number of lung metastatic nodules. *P*, by Mann–Whitney *U*-test. (**c**) Representative images of pleural metastasis of animals injected intravenously with control SW620 cells. (**d**) Quantification of the pleural nodules in each experimental group. *P*, by Mann–Whitney *U*-test. (**e**) Representative H&E images of pleural invasion and costal cartilage invasion tissue were shown, respectively. Scale bars, 6 mm (right) and 800 μm (left)

Considering the role of IGFBP-rP1 in controlling EMT and migration *in vitro*, we extended our study to an immunocompetent mouse model of distant metastasis. IGFBP-rP1 overexpressing SW620 cells led to a significant reduction of lung metastasis compared with scramble control cells, as shown in the gross images (Figure 4a, left) and hematoxylin and eosin (H&E) staining (Figure 4a, right). Decreased numbers of lung metastatic nodules were quantified as shown in Figure 4b. Interestingly, we also observed less probability of metastatic lesion formation and decreased nodule numbers in the pleura of IGFBP-rP1 overexpressing group when compared with control group (Figures 4c and d). H&E staining indicated tumor cells invaded the pleural (Figure 4e, left top) and even the costal cartilage (Figure 4e, left bottom) in the group without IGFBP-rP1 expression. Therefore, these analyses again show that IGFBP-rP1 strongly opposes colorectal cancer distant metastasis.

### Effect of IGFBP-rP1 on TGF-*β* receptor and its downstream mediators

**Figure 5:**
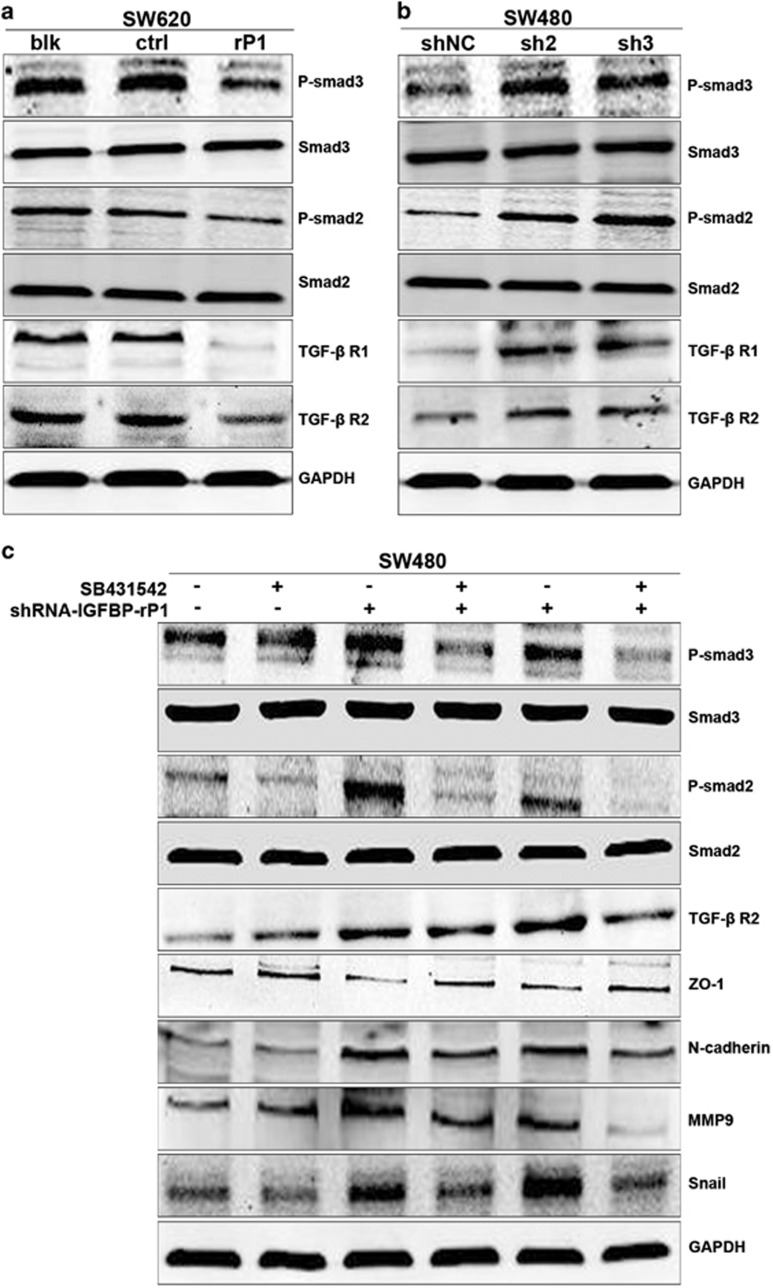
IGFBP-rP1 inhibits TGF-*β* receptor expression and its downstream signaling in SW620 and SW480 cells. (**a**) SW620 cell lysates from blk, vector, and IGFBP-rP1 cells were analyzed the indicated proteins by western blot. (**b**) SW480 cell lysates from scramble shRNA and IGFBP-rP1 shRNA cells were analyzed the indicated proteins by western blot. (**c**) Western blot of the indicated antibodies in two SW480-IGFBP-rP1 knockdown cell clones and controls untreated or treated with 10 ng/ml SB431542 for 48 h

TGF-*β* is considered as a very potent inducer of EMT and metastasis. Canonical Smad signaling, which is activated by TGF-*β*, has critical roles during the induction of EMT.^22, 23^ Previous microarray results revealed a relationship between IGFBP-rP1 and TGF-*β* signaling, especially Smad3. Therefore, we explored whether the effect of IGFBP-rP1 on EMT is mediated by TGF-*β* signaling. As expected, IGFBP-rP1 overexpression inactivated both TGF-*β* R I and R II in SW620 cells. Accordingly, phosphorylation of smad2 and smad3 was also decreased in the IGFBP-rP1 overexpressing cells as compared with the control cells (Figure 5a). In support of this, IGFBP-rP1 knockdown in SW480 cells increased the levels of TGF-*β* receptor and its main downstream effectors, phospho-Smad2/3 (Figure 5b). To further validate whether IGFBP-rP1 modulated the TGF-*β*/Smad signaling pathway, we used SB431542, which is a selective inhibitor of TGF-*β*/ALK5/Smad2 signaling. Western blots confirmed the inhibition of TGF-*β* R II and smad2/3 phosphorylation by SB431542 in two clones of IGFBP-rP1 knockdown SW480 cells. SB431542 rescued the EMT status induced by IGFBP-rP1 knockdown (Figure 5c). Overall, our data support that IGFBP-rP1 upregulates TGF-*β* receptor expression, with subsequent activation of TGF-*β* receptor pathways.

### IGFBP-rP1 reverses TGF-*β*1-mediated EMT by attenuating Smad pathways

**Figure 6:**
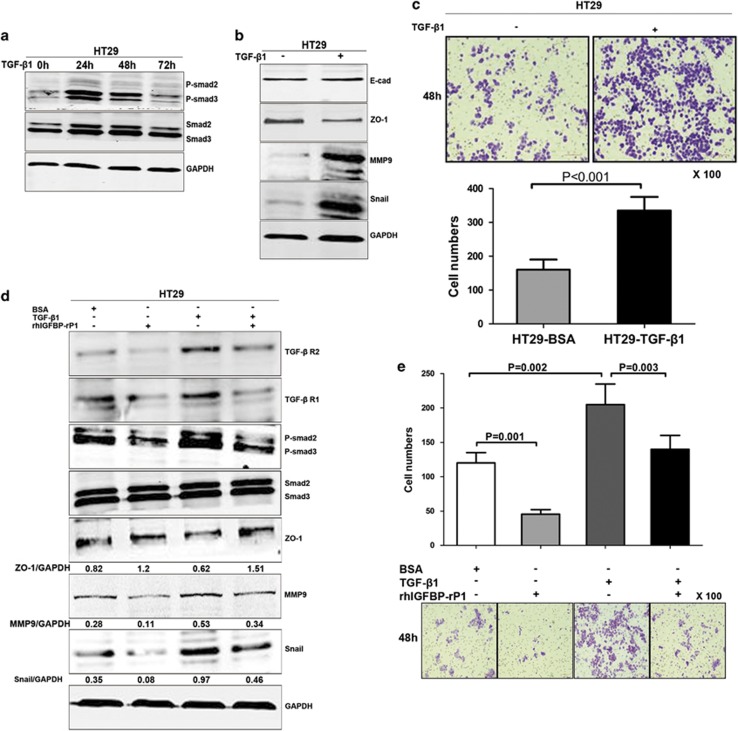
IGFBP-rP1 blocks TGF-*β*1-induced EMT in HT29 cells. (**a**) HT29 cells were incubated with TGF-*β*1 (10 ng/ml) for the indicated times. The levels of P-smad2/3 were assessed by western blot. (**b**) Expression of EMT markers assessed by western blot. (**c**) TGF-*β*1 promoted cell migration determined by transwell migration assay. The results were shown as mean+S.D. from triplicate experiments. (**d**, **e**) Western blot of the indicated antibodies and transwell migration assay in HT29 cells and controls untreated or treated with 1 μg/ml rhIGFBP-rP1 and/or 10 ng/ml TGF-*β*1. The results were shown as mean+S.D. from triplicate experiments

The relevance of the pathway was next examined in HT29 colorectal cancer cells. Here, addition of TGF-*β*1 induced a time-dependent increase of phosphorylated Smad2/3 protein levels, with 24 h TGF-*β* having the maximal activation effect in HT29 cells (Figure 6a). In response to TGF-*β*1 stimulation for 24 h, although the level of E-cadherin showed no apparent change, HT29 cells underwent EMT as determined by downregulation of the epithelial marker ZO-1 with concomitant upregulation of MMP9 and Snail (Figure 6b) as well as increased cell migration (Figure 6c). These data suggested that we had not only established the classical model of TGF-*β*1-induced EMT, but that it also activated the TGF-*β*/Smad cascade in HT29 cells. To further evaluate whether IGFBP-rP1 reversed TGF-*β*1-induced EMT-related changes, we performed a rescue experiment by adding exogenous rhIGFBP-rP1 protein. Consistent with our findings in the other cell lines, treatment with rhIGFBP-rP1 alone decreased TGF-*β* receptor and phospho-Smad2/3 expression levels as well as inhibited EMT program (Figure 6d, lane 2). This was accompanied by an obvious decrease of HT29 cells invasive ability (Figure 6e, lane 2). Treatment with TGF-*β* alone activated EMT program, TGF-*β* receptors, and its downstream molecules (Figure 6d, lane 3). Of note, exogenous rhIGFBP-rP1 protein decreased the activation of TGF-*β* signaling and restored the upregulation of TGF-*β*1-induced EMT-related markers (Figure 6d, lane 4 compare with lane 3). Accordingly, rhIGFBP-rP1 decreased the promotion of migration triggered by TGF-*β*1 (Figure 6e). These data collectively provide evidence that IGFBP-rP1 suppresses EMT by attenuation of TGF-*β*/Smad signaling in colorectal cancer.

## Discussion

IGFBP-rP1 is commonly regarded as a tumor suppressor in CRC,^20^ prostate cancer,^15^ breast cancer,^16^ lung cancer,^17^ melanoma,^18^ and thyroid cancer.^19^ Previous work from our laboratory suggested that IGFBP-rP1 expression in CRC tissue is correlated with a favorable prognosis.^20^ Although the prognosis relies heavily on tumor recurrence and distant metastasis after surgical resection, little was known about the function of IGFBP-rP1 in tumor cell motility.

Although not well investigated, IGFBP-rP1 seems to have a role in cell migration. Downregulation of its expression promotes invasion by hepatocellular cancer cells.^24^ IGFBP-rP1-overexpressing cells in breast cancer show reduced migration,^25^ and IGFBP-rP1 is also correlated with early metastasis of CRC.^26, 27^ We previously demonstrated that the restoration of IGFBP-rP1 by 5-aza-dC inhibits migration and invasion in CRC cell lines. Consistent with these results, the present study demonstrated that depletion of IGFBP-rP1 promoted the migration of SW480 cells, whereas its overexpression inhibited CRC cell migration *in vitro* and distant metastasis *in vivo*. As expected, the TGF-*β*-induced migration was reversed by rhIGFBP-rP1. Moreover, downregulation of IGFBP-rP1 occurred in patients with positive lymph-node metastasis. Overall, IGFBP-rP1 showed an inhibitory effect on cell motility and tumor metastasis in CRC.

EMT is a critical process for cancer metastasis. Until recently, IGFBP-rP1 had not been associated with EMT regulation. In this report, we demonstrated the role of IGFBP-rP1 in regulating EMT. Our study choosed SW620 and CW2 cell lines, the two colon cancer cell lines without endogenous IGFBP-rP1 expression, to observe the transfection of IGFBP-rP1 cDNA on the EMT status of the cells. SW480 cells that were used shRNA to stably knock down express endogenous IGFBP-rP1. HT29 is a widely used colorectal cell line for a classical model system: TGF1-induced EMT. We used a combination of *in vitro* cell-line models and *in vivo* xenograft nude mice to show that gain of IGFBP-rP1 facilitated the acquisition of numerous epithelial features and the absence of mesenchymal characteristics. Conversely, loss of IGFBP-rP1 induced apparent EMT. Although the decrease of IGFBP-rP1-mediated E-cadherin expression is not obvious in HT29 cells, we found that E-cadherin was induced in overexpressing SW620 and CW2 cells compared with the control vector transfected cells and the parental cells. Moreover, another epithelial marker ZO-1 in SW480 and HT29 cells was investigated. E-cadherin that is a transmembrane protein represents a well-characterized molecular marker of the epithelial phenotype. Numerous studies have demonstrated that abolishment of E-cadherin expression or function could promote cell invasiveness, which is a critical event in EMT. However, E-cadherin losing is not a unique pivotal event during EMT. The forcing expression of E-cadherin was not sufficient to restore epithelial characteristic in murine spindle carcinoma cells^28^ as well as failed to reverse the fibroblastic morphology of MDCK-Snail cells.^29^ Furthermore, reduced E-cadherin expression triggered by overexpression of Slug and Twist failed to induce full EMT and enhance metastasis in CRC cells.^30^ More importantly, clinical data analyses also indicated that IGFBP-rP1 is inversely associated with the classical EMT markers. These data indicate that IGFBP-rP1 may be a negative regulator to monitor the EMT program.

IGFBP-rP1 is known to bind to IGF-1 and IGF-2 with 100-fold lower affinity than IGFBP1 to IGFBP6,^31^ so it functions in both an IGF-dependent and an IGF-independent manner.^32^ IGFBP-rP1 may be involved in the TGF-*β* signaling pathway through binding to activin,^33^ a member of the TGF-*β* superfamily. IGFBP-rP1 in prostate epithelial cells is also modulated by TGF-*β*1.^34^ Previous microarray analysis provided evidence that IGFBP-rP1 has a close relationship with TGF-*β*, and further influences Smad3 expression.^35^ Therefore, whether IGFBP-rP1 participates in TGF-*β* signaling by a Smad-dependent or Smad-independent manner remains to be confirmed. Interestingly, we found that TGF-*β* signaling inhibition was due to overexpression of IGFBP-rP1 levels in the SW620 cells. Reversely, knockdown of IGFBP-rP1 in SW480 cells activated the TGF-*β* signaling pathway. To strengthen our data and provide further insight, exogenous rhIGFBP-rP1 protein decreased the absence of TGF-*β* receptor and its downstream protein levels activated by TGF-*β*1 in HT29 cells. EMT as a potential target for IGFBP-rP1 *via* regulation of TGF-*β* /Smad signaling is a unique finding based on our studies. In the study, we delineate a pathway in which IGFBP-rP1 attenuates the TGF-*β* signaling pathway to suppress EMT, thereby decreasing migration.

TGF-*β* exerts a potent effect on EMT initiation and tumor metastasis. It has been reported that TGF-*β* induces the expression of transcription factors such as Snail, Slug, Zeb, and Twist.^10^ Of these, Snail is greatly upregulated through the Smad pathway.^36^ During TGF-*β*-mediated EMT, the Snail-Smad3/4 transcriptional complex functions as a transcriptional repressor.^37^ HMGA2 and Smads bind to the Snail promoter and acquire the EMT phenotype.^38^ These compelling data suggest that Snail is regulated by TGF-*β*/Smad signaling from the tumor microenvironment. In line with these observations, Snail was clearly downregulated by either IGFBP-rP1 (Supplementary Figure 1B) or the addition of rhIGFBP-rP1 to TGF-*β*1-stimulated cells (Figure 6d) and was upregulated by IGFBP-rP1 knockdown (Supplementary Figure 1C). Therefore, the potential involvement of the IGFBP-rP1-Smad-Snail axis in CRC warrants further investigation.

In brief summary, our analyses have concluded that IGFBP-rP1, through the inhibition of TGF-*β*/Smad signaling, endows cells with properties linked to metastasis, including a reverse EMT process and decreased motility.

## Materials and methods

### Reagents/Materials

Cell-culture reagents were from Gibco (Grand Island, NY, USA). Lipofectamine and TRIzol were from Invitrogen (Carlsbad, CA, USA). G418 was from Merck (Darmstadt, Germany). Monoclonal antibody to IGFBP-rP1 and recombinant human IGFBP-rP1 protein were from RD (Minneapolis, MN, USA). Mouse monoclonal antibodies to E-cadherin, N-cadherin, TGF-*β* R I, TGF-*β* R II, *β*-catenin, actin, and glyceraldehyde 3-phosphate dehydrogenase (GAPDH) were from Santa Cruz Biotechnology (Santa Cruz, CA, USA). Polyclonal antibodies to vimentin, MMP9, Snail, Smad2, P-smad2, Smad3, and P-smad3 were from Cell Signaling (Danvers, MA, USA). Recombinant human TGF-*β*1 was from Peprotech (Rocky Hill, NJ, USA). SB431542 (TGF-*β*/ALK5/Smad2 inhibitor) was obtained from Sigma.

### Cell culture

The human CRC cell lines SW620 and SW480 were purchased from the American Type Culture Collection (Manassas, VA, USA). The human CRC cell lines HT29 and CW2 were obtained from the Shanghai Institute of Cell Biology, Chinese Academy of Science (Shanghai, China). All cell lines were maintained in RPMI-1640 supplemented with 10% fetal bovine serum (FBS) at 37 °C, in a humidified atmosphere of 5% CO_2_. The medium was changed every 2 days. The stably-transfected cell lines SW620-ctrl/rP1 and CW2-ctrl/rP1 had been established previously.^20, 39^

### Transfection

The IGFBP-rP1-shRNA expression vector kit was from Shanghai Genepharma Co., Ltd. (Shanghai, China), and the target sequences were TAGAGGAGATACCAGCACCCAGCCA and TGCTGATGCTGAAGCCTGTCCTTGG. All transfection experiments were performed using Lipofectamine 2000 (Invitrogen) according to the manufacturer's protocol. Stably-transfected cell clones were selected using G418 (500 *μ*g/ml).

### RNA isolation and real-time RT-PCR

Total RNA was extracted from cells using TRIzol reagent (Invitrogen) according to the manufacturer's protocol. One microgram of total RNA was reverse transcribed using a PrimeScript RT reagent kit (Perfect Real Time) and real-time PCR was performed using SYBR Premix Ex Taq (Tli RNaseH Plus), both from TaKaRa Biotechnology Co., Ltd (Dalian, China). The 10-*μ*l reactions were performed in 384-well plates on the ABI Prism 7900 sequence detection system (Applied Biosystems, Foster City, CA, USA). The settings were initial denaturation at 95 °C for 10 s, followed by 40 cycles of 95 °C for 5 s and 60 °C for 30 s, and elongation at 72 °C for 40 s. For the normalized expression ratios of genes, the *△△*Ct method was used. The primer sequences used were as follows: GAPDH, forward: 5′-ACCACAGTCCATGCCATCAC-3′ and reverse: 5′-TCCACCACCCTGTTGCTGTA-3′ IGFBP-rP1, forward: 5′-CACTGGTGCCCAGGTGTACT-3′ and reverse: 5′-TTGGATGCATGGCACTCATA-3′.

### Western blot analysis

Concentrated cell culture medium and total cell proteins were prepared as described previously. Western blot analyses were performed using standard procedures. Proteins were separated by SDS-PAGE and transferred onto nitrocellulose membrane. After blocking in 5% non-fat milk in TBS-Tween 20 for 30 min at room temperature, membranes were incubated with primary antibodies overnight at 4 °C, and then incubated with secondary antibodies for 1 h at room temperature. The blots were detected using the Odyssey system (Li-COR, Lincoln, NE, USA). Primary antibodies directed against E-cadherin (1 : 500), N-cadherin (1 : 500), vimentin (1 : 1000), MMP9 (1 : 1000), Snail (1 : 1000), TGF-*β* R I (1 : 200), TGF-*β* R II (1 : 200), Smad2 (1 : 1000), P-smad2 (1 : 1000), Smad3 (1 : 1000), P-smad3 (1 : 1000), GAPDH (1 : 5000), and actin (1 : 5000) were used in western blot analyses. GAPDH and actin served as loading controls. The experiments were repeated at least three times.

### Recombinant human IGFBP-rP1 protein treatment

Recombinant human IGFBP-rP1 (rhIGFBP-rP1) protein stimulation assays were done as described previously. Cells were seeded into 6-well plates (3–5 × 10^5^cells/plate). After attachment for 24 h, the cells were exposed to fresh culture medium containing different concentrations of rhIGFBP-rP1 protein (SW620 cells, 4 μg/ml; HT29 cells, 1 μg/ml). After 48 h, the cells were collected and protein extraction performed.

### Recombinant human TGF-*β*1 treatment

HT29 cells were grown to 70% confluence and exposed to serum-free medium. After starvation for 24 h, the cells were incubated with recombinant human TGF-*β*1 at 10ng/ml for the indicated periods. EMT markers were analyzed by western blot.

### Phalloidin staining

Cells were grown on glass slides, washed three times with PBS, and fixed in 100% methanol for 10 min at −20 °C. They were washed again with PBS, permeabilized with 0.1% Triton X-100 in PBS for 15 min, and blocked in 10% bovine serum for 30 min at room temperature. After further washing with PBS, the cells were incubated with primary antibody Alexa-Fluor 488 phalloidin (1 : 1000; Invitrogen) for 2 h in the dark at room temperature, and washed with PBS. The nuclei were stained with 4',6-diamidino-2-phenylindole (1 : 10000; Invitrogen) for 30 min. Confocal images were captured using an LSM 510 META confocal microscope (Carl Zeiss, Oberkochen, Germany).

### Immunofluorescence

Glass slides prepared as mentioned above. The cells were incubated with mouse anti-E-cadherin and anti-*β*-catenin (1 : 50; Santa Cruz) antibodies at 4 °C overnight followed by washing with PBS three times. Coverslips were then incubated with Texas Red-conjugated anti-mouse antibodies (1 : 200; Invitrogen) for 30 min at room temperature, then stained with 6-diamidino-2-phenylindole (1 : 10 000; Invitrogen).

### Wound-healing assay

Cells were grown to ~80% confluence in 6-well plates. A straight scratch was made gently across the center of the well using a yellow Eppendorf tip. Detached cells were rinsed away with PBS and serum-free medium was added. Cell migration to the scratch was monitored at the indicated time periods in the same area. The images were captured at × 100 magnification (Nikon TS100, Tokyo, Japan). The experiments were repeated three times.

### Transwell migration assay

Boyden chambers (8 *μ*m pores; Costar, Corning, NK, USA) were used for transwell assays to measure cell migration. Cells (1 × 10^5^) in 0.2 ml serum-free medium were seeded into the upper chamber, while 0.6 ml medium containing 10% FBS was added to the lower chamber. The cells were then incubated for 48 h at 37 °C in a humidified atmosphere of 5% CO_2_. After the assay was completed, the cells on the upper side of the membrane were removed with a wet cotton swab and those on the lower side were fixed in 95% ethanol and stained with crystal violet at room temperature for 10 min. The membrane was gently removed and mounted on a glass slide. The images were captured at × 100 magnification. The number of cells was counted from four random fields using ImageJ (NIH, Bethesda, MD, USA). Each experiment was performed at least three times.

### Tumor formation assay

BALB/c-nu/nu mice (5 weeks old, male) were purchased from Shanghai Laboratory Animal Center. All experimental procedures were approved by the Animal Care and Use Committee of Zhejiang Chinese Medical University. Cells were harvested and suspended in PBS. Control vector overexpressing SW620 cells and IGFBP-rP1 overexpressing SW620 cells (3 × 10^6^ cells; *n*=4 mice each); control shRNA SW480 cells, IGFBP-rP1 knockdown SW480 cells (8 × 10^6^ cells; *n*=4 mice each) in 0.1 ml PBS were injected subcutaneously into the nape of mice. The length and width of the tumor were measured using digital calipers twice a week until the end point. Tumor volume was calculated using the following formula: π/6 × (length) × (width)^2^. After 6–7 weeks, the mice were euthanized, and the tumors were excised, weighed, and then used for RNA and protein extraction.

### Tumor metastasis assays

NOD/SCID mice (5 weeks old, male) were purchased from Shanghai Laboratory Animal Center. To determine the distant metastatic potential of cancer cells *in vivo*, 5 × 10^6^ cells (two groups including SW620-vector cells and SW620-IGFBP-rP1 cells) resuspended in 100 μl PBS were injected into the tail vein of severe combined immunodeficient NOD/SCID mice (*n*=4/group). After 6 weeks, the number of metastatic lesions on the lung and pleura was counted. Individual organs were excised and analyzed by hematoxylin and eosin (H&E) staining.

### Clinical specimens

All 217 patients were enrolled with informed consent in accordance with the Review Boards of Zhejiang University. Data of the clinicopathological parameters were obtained from the Xiaoshan tumor registry system database. The 217 specimens were from 117 males and 100 females. The age at diagnosis ranged from 26 to 85 years (median, 61) and follow-up ranged from 1 to 207 months (median, 95).

### Immunohistochemical staining and scoring

Immunohistochemistry was performed using the EnVision method as previously described.^40^ Antibodies, methods, dilutions, and staining pattern are listed in Supplementary Table S1. Each specimen was scored as follows: 0, negative or <5% 1, 5–25% 2, 26–50% 3, 51–75% 4, >75% positive cells. Subsequently, the cases were classified into low (scores 0–3) and high expression (score 4) of IGFBP-rP1. For E-cadherin, *β*-catenin, and fibronectin, the scoring was negative (<5%) or positive (≥5%).

### Statistical analysis

The statistical package SPSS (version 20.0; IBM, New York, NY, USA) was applied. For results from cell lines, all data are reported as mean±S.D. Unpaired Student's *t* tests were used for normally distributed data and non-parametric Mann–Whitney *U*-tests were used for non-normally distributed data to compare central tendencies. For results in CRC tissues, comparisons of clinicopathological parameters and EMT markers in the IGFBP-rP1-low and IGFBP-rP1-high groups were made by the *χ*^2^ test or Fisher's exact test. Correlations were analyzed by the Spearman coefficient test. Significance was set at *P*<0.05.

## Figures and Tables

**Table 1:** Correlation between the expression of IGFBP-rP1 immunohistochemical staining and clinicopathological characteristics in colorectal cancer

**Variable**	**Total (*n*)**	**IGFBP-rP1 expression**		***r***	***P*-value**
		**Low (*n* (%))**	**High (*n* (%))**		
*Age (years)*					
<60	104	20 (19.2)	84 (80.8)	−0.046	0.496
≥60	113	26 (23.0)	87 (77.0)		
					
*Sex*					
Male	117	25 (21.4)	92 (78.6)	0.004	0.947
Female	100	21 (21.0)	79 (79.0)		
					
*Histologic grade*					
Low	145	24 (16.6)	121 (83.4)	−0.161	0.017*
High	72	22 (30.6)	50 (69.4)		
					
*Lymph-node metastasis*					
Negative	121	18 (14.9)	103 (85.1)	−0.174	0.011*
Positive	96	28 (29.2)	68 (70.8)		
					
*Distant metastasis*					
Negative	195	42 (21.5)	153 (78.5)	0.025	0.715
Positive	22	4 (18.2)	18 (81.8)		
					
*TNM stage*					
I-II	116	18 (15.5)	98 (84.5)	−0.149	0.028*
III-IV	101	28 (27.7)	73 (72.3)		

*P<0.05, *χ*^2^ test

*r*, Spearman rank correlation test

**Table 2:** Correlation between the expression of IGFBP-rP1 immunohistochemical staining and EMT markers in colorectal cancer

**Variable**	**Total (*n*)**	**IGFBP-rP1 expression**		***r***	***P*-value**
		**Low (*n* (%))**	**High (*n* (%))**		
*E-cadherin membrane expression*					
Negative	47	16 (34.0)	31 (66.0)	0.240	0.014*
Positive	58	8 (13.8)	50 (86.2)		
					
*β-Catenin membrane expression*					
Negative	72	24(33.3)	48 (66.7)	0.280	0.002*
Positive	53	5(9.4)	48 (90.6)		
					
*Fibronectin expression*					
Negative	40	5 (12.5)	35 (87.5)	−0.196	0.030*
Positive	82	25 (30.5)	57 (69.5)		

*P<0.05, *χ*^2^ test

*r*, Spearman rank correlation test

## Abbreviations

IGFBP-rP1: Insulin-like growth factor-binding protein-related protein 1
CRC: colorectal cancer
EMT: epithelial–mesenchymal transition
TGF-*β*: transforming growth factor-*β*
shRNA: short hairpin RNA
blk: blank
ctrl: control
NC: negative control
