# Supplementary material for: IGFBP-rP1 suppresses epithelial–mesenchymal transition and metastasis in colorectal cancer
Source: Cell Death Dis. 2015 Mar 19;6(3):e1695–. doi: 10.1038/cddis.2015.59 (PMC4385937; doi:10.1038/cddis.2015.59)
Supplement: Supplementary Figure S1 [file cddis201559x1.doc]

**Supplementary Figure S1**

**
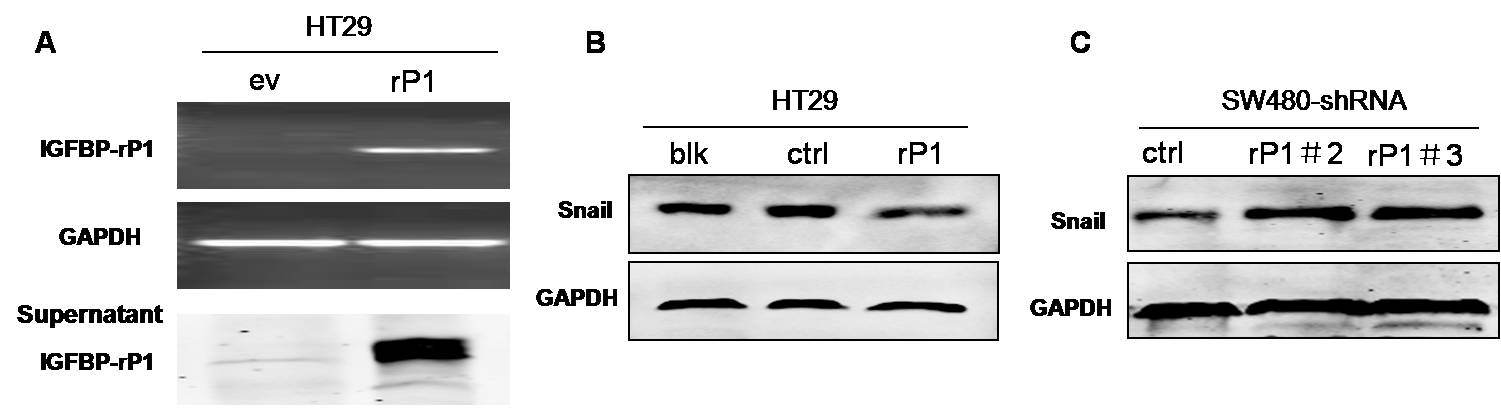
**

**Supplementary Figure S1 IGFBP-rP1 inhibits Snail expression. (A)** The transfection efficiency was confirmed by RT-PCR and western blot analyses. **(B)** The level of Snail in HT29-IGFBP-rP1 cells assessed by western blot. **(C)** The level of Snail in IGFBP-rP1 knockdown SW480 cells assessed by western blot.
