# Supplementary material for: IGFBP-rP1 suppresses epithelial–mesenchymal transition and metastasis in colorectal cancer
Source: Cell Death Dis. 2015 Mar 19;6(3):e1695–. doi: 10.1038/cddis.2015.59 (PMC4385937; doi:10.1038/cddis.2015.59)
Supplement: Supplementary Table S1 [file cddis201559x2.doc]

| **Supplementary Table S1. Antibodies and immunostaining methods** | | | | | | | | |
| --- | --- | --- | --- | --- | --- | --- | --- | --- |
| **Antibody** |  | **Source** |  | **Antigen retrieval** |  | **Dilution** |  | **Staining pattern** |
| E-cadherin |  | Santa Cruz |  | Microwave, citrate buffer (pH 6.0), 20 min |  | 1:200 |  | membranal |
| β-catenin |  | Maixin |  | Microwave, citrate buffer (pH 6.0), 30 min |  | 1:200 |  | membranal |
| Fibronectin |  | Santa Cruz |  | Pancreatic enzyme, 37 ℃, 30 min |  | 1:50 |  | Cytoplasmic |
| IGFBP-rP1 |  | Santa Cruz |  | Microwave, citrate buffer (pH 6.0), 20 min |  | 1:200 |  | Cytoplasmic |
